# Stoichiometric Responses of Soil Microbes and Enzymes to Altitudinal Gradients in Alpine Meadows

**DOI:** 10.3390/microorganisms13122692

**Published:** 2025-11-25

**Authors:** Yongqian Li, Jun Wu, Wenjun Ma, Zhengqian Zhou, Hongming Zhang, Liqun Cai, Dong Lin

**Affiliations:** 1 College of Resources and Environment, Gansu Agricultural University, Lanzhou 730070, China; 2 State Key Laboratory of Aridland Crop Science, Gansu Agricultural University, Lanzhou 730070, China; 3 College of Forestry, Gansu Agricultural University, Lanzhou 730070, China; 4 College of Grassland Science, Gansu Agricultural University, Lanzhou 730070, China

**Keywords:** Qinghai–Tibet Plateau, ecological stoichiometry, soil extracellular enzymes, microorganism nutrient limitation

## Abstract

Soil microbial nutrient limitation is of great significance for the maintenance of soil fertility, the sustainability of plant growth, and the stability of the alpine meadow ecosystems, which are particularly sensitive to global climate change. This study aimed to explore the effects of soil extracellular enzyme activities on soil microbial nutrient limitation across three altitudinal gradients—low altitude (LA: 2900–3200 m above sea level (masl)), middle altitude (MA: 3200–3500 masl), and high altitude (HA: 3500–3800 masl)—in alpine meadows in the northeastern Qinghai–Tibet Plateau, using the method of ecological stoichiometry. The research results showed that soil nutrients mostly accumulate in the surface layer: with increasing altitude, soil organic carbon (SOC) and total nitrogen (TN) contents gradually increase (*p* < 0.05), and their contents at high altitude in the 0–20 cm soil layer are twice those at low altitude. The activities of β-1,4-glucosidase (BG) and β-1,4-N-acetylglucosaminidase (NAG) at high altitude are significantly 26.77% and 30.88% higher than those at low altitude, respectively. Linear regression analysis shows a significant positive correlation between soil nutrients and C/N/P-related enzymes after logarithmic transformation along the altitudinal gradient. Enzyme vector analysis revealed that in the alpine meadows at altitudes ranging from 2900 to 3800 masl, relative nitrogen limitation was widespread, while relative carbon limitation was more significant in both high-altitude and low-altitude regions (*p* < 0.05). Notably, this study did not account for the granulometric composition of the soil at the sampling sites. Nevertheless, it partially reveals the nutrient acquisition strategies of microorganisms across different altitudinal gradients, providing a theoretical basis for understanding nutrient cycling in alpine meadow ecosystems and addressing global change.

## 1. Introduction

As a key environmental filtering factor in alpine meadow ecosystems, the altitudinal gradient constructs soil ecosystems with vertical differentiation characteristics by driving synergistic changes in air temperature, precipitation, vegetation types, and soil development [1]. With increasing altitude, the mean annual temperature decreases at a rate of approximately 0.6 °C per 100 m, and the precipitation pattern shows a unimodal distribution [2]. Vegetation successions range from drought-tolerant herbs at low altitudes to cold-tolerant shrubs and coniferous forests at high altitudes [3]. This multi-dimensional environmental gradient may directly expose soil microbial communities to the dual stress of increased litter C/N ratio and reduced root exudates, thereby triggering the functional reorganization of soil extracellular enzyme activities and adaptive adjustments of stoichiometric characteristics [4]. Through the synergistic effects of climate–vegetation–microorganisms [5], the altitudinal gradient shapes the functional pattern of extracellular enzymes, which in turn regulates the release rate, form, and availability of soil nutrients [6]. With the rise in carbon dioxide concentration, alpine meadows will accelerate their soil carbon cycle under different nitrogen additions by stimulating microbial activities rather than reducing microbial metabolic efficiency [7]. The reduction in carbon allocation to roots leads to a decrease in total soil organic carbon, which may affect carbon sequestration in semi-natural grasslands under elevated ozone conditions [8]. This vertical differentiation of soil ecosystems driven by altitude is not only a core process of biogeochemical cycles, but also a key entry point for understanding the maintenance mechanism of carbon sink stability under the background of global warming [9].

As components of soil ecosystems, soil microorganisms directly regulate the synthesis and secretion processes of extracellular enzymes through their community composition and metabolic activity [10]. By secreting various enzymes (e.g., β-1,4-glucosidase (BG), β-1,4-N-acetylglucosaminidase (NAG), β-1,4-cellobiohydrolase (CBH), leucine aminopeptidase (LAP), and acid (alkaline) phosphatase (AP)), soil microorganisms drive the decomposition of soil organic matter and nutrient cycling. Some bacterial taxa such as *Staphylococcus aureus* rely on enzymes such as leucine aminopeptidase (LAP) to release nitrogen (N) for meeting their growth demands [11]. Fungi-dominated communities tend to secrete oxidases to decompose complex carbon (C) substrates such as lignin [12], while bacteria-dominated communities accelerate nitrogen turnover via high-activity proteases [13]. Under the combined conditions of increased precipitation and nitrogen deposition, the proportion of soil fungi decreases, and the microbial community composition may shift toward bacterial dominance [14]. This functional division is essentially a reflection of microorganisms’ strategy to adapt to the stoichiometry of soil resources: when soil is carbon-limited, microorganisms upregulate cellulase activity to enhance carbon acquisition [15]; in response to phosphorus deficiency, the secretion of phosphatase can increase by 2–3 times to promote the release of inorganic phosphorus, driving phosphorus mineralization and dissolution [16]. Additionally, the Microbial Metabolic Theory [17] predicts that extracellular enzyme activity has a power-law correlation with the microbial biomass carbon-to-nitrogen ratio [18,19]. This inherent coupling relationship not only determines the kinetic characteristics of enzymatic reactions but also maintains the material cycling efficiency of soil ecosystems through the coevolution of the “microorganism-enzyme” functional module [20]. This functional coupling between microorganisms and enzymes constitutes the core biological mechanism of soil nutrient cycling [21], and plays an important role in understanding the nutrient cycling of microorganisms along altitudinal gradients.

As the nutrient cycling benchmark of soil ecosystems, stoichiometric ratios provide a unique perspective for accurately analyzing the dynamics of soil nutrient cycling. The stoichiometric ratios of key elements in soil can directly reflect the availability of soil nutrients, turnover rate, and microbial metabolic strategies [22]. In recent years, enzyme stoichiometry models have been proposed to indicate soil microbial nutrient limitation, among which the enzyme vector analysis model is a method in microbial ecology for quantifying the microbial demand for nutrients such as carbon (C), nitrogen (N), and phosphorus (P) [23]. This method calculates the C/N, C/P, and N/P ratios of enzyme activities to generate vector length (VL) and vector angle (VA) of soil extracellular enzyme stoichiometry. Relative to nutrient demand, an increase in VL indicates an increase in relative C limitation, while a VA greater than 45° suggests the potential existence of P limitation. Thus, it reveals the relative demand and preference of microbial communities for nutrients, and predicts nutrient limitation to a certain extent [24]. These stoichiometric properties not only clarify the balance between supply and demand of soil nutrients, but also indicate the nutrient utilization strategies of microbial communities and the regulatory direction of extracellular enzyme activities [25]. During growth and metabolism, microorganisms should keep a specific elemental composition ratio to meet physiological demands. When the soil nutrient stoichiometric ratio deviates from microbial requirements, microorganisms are expected to adjust the secretion pattern of extracellular enzymes [26]. For example, in P-deficient soils, microorganisms increase phosphatase activity to accelerate the mineralization of organic phosphorus, thereby alleviating P limitation [27]. This regulation of enzyme activity based on nutrient supply and demand is essentially achieved by altering the enzyme stoichiometric ratio. The dynamic changes in soil stoichiometric ratios do not exist in isolation; their association with environmental gradients is equally close. Furthermore, variations in stoichiometric ratios along different altitudinal gradients can map the impact pathways of environmental factors such as climate and vegetation on soil nutrient cycling [28].

Although the impacts of altitudinal gradients on soil ecosystems and the associations between microorganisms and extracellular enzyme activities have received widespread attention, the specific effects of soil nutrients on the stability characteristics of soil microorganisms and nutrient resource limitations in meadow ecosystems remain unclear. To address this issue, we propose the following hypotheses: (1) As the altitudinal gradient gradually increases, decreasing temperatures affect organic matter content and alter the stoichiometric ratios of soil extracellular enzymes; simultaneously, microorganisms exhibit a more pronounced trend toward being dominated by carbon-acquiring enzymes. (2) Soil nutrients and extracellular enzyme activities are mainly limited by nitrogen, and microorganisms can maintain a certain level of stability by adjusting their enzyme secretion strategies. This study aims to explore the impact patterns of altitude on soil extracellular enzyme activities and stoichiometry in the top 20 cm of soil on the Qinghai–Tibet Plateau, as well as the key elements limiting soil nutrient absorption.

## 2. Materials and Methods

### 2.1. Description of the Study Area and Site

This study was conducted in Luqu County, Gannan Tibetan Autonomous Prefecture, Gansu Province, China (33°57′–34°48′ N, 101°36′–102°58′ E, the altitudinal range is 2827–4366 m above sea level (masl)). Located in the Qinghai–Tibet Plateau climatic zone, this area belongs to the alpine humid climate region. The mean annual temperature is 2.3 °C, with mean annual precipitation ranging from 633 to 782 mm; the monthly mean maximum temperature is 13.1 °C, and the monthly mean minimum temperature is −8.1 °C. The frost-free period lasts for approximately 30 days, and the average depth of frozen soil is about 1.4 m. The study area is situated in the alpine forest-steppe plant zone, encompassing five vegetation types—desert, meadow, shrub, forest, and meadow steppe—among which meadow is the vegetation type with the largest coverage area. In this study, 21 sampling sites were selected in subalpine meadows and shrubby meadows. Influenced by the plateau and mountain climates, the soil exhibits high diversity; moreover, with the change in altitude, soil types show a regular vertical variation. The soils in this area can be classified into five major categories—subalpine meadow soil, gray-brown soil, dark meadow soil, peat soil, and marsh soil—with subalpine meadow soil being the dominant type. The main plant species composition and latitude/longitude coordinates of each altitude are summarized in Table 1.

### 2.2. Study Design and Field Sampling

**Figure 1:**
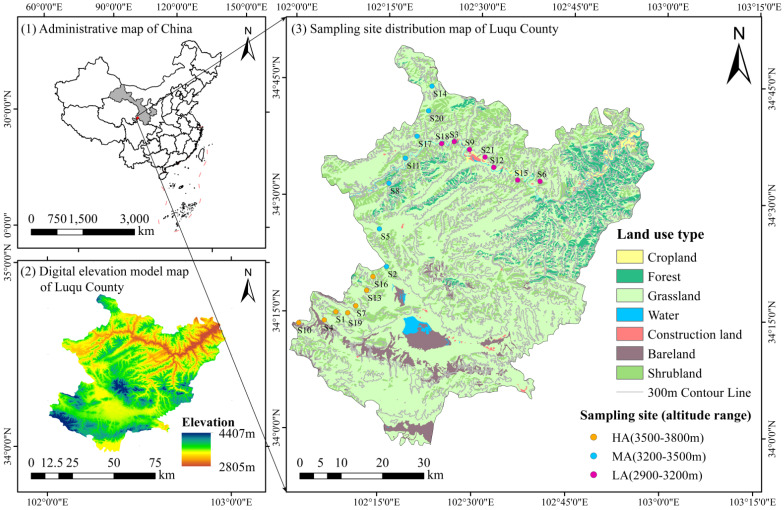
Location of the study area. LA: low altitude; MA: middle altitude; HA: high altitude.

Field investigation and soil sampling were conducted in August 2024. 7 sampling sites were selected for each of the 3 altitudinal gradients (LA: 2900–3200 masl, MA: 3200–3500 masl, HA: 3500–3800 masl), and 3 independent plots were randomly chosen as biological replicates (n = 3) for each site. The locations of the sampling sites are shown in Figure 1. Soil samples were collected from two soil depth layers (0–10 cm and 10–20 cm), and the activities of β-1,4-glucosidase (BG), β-1,4-cellobiohydrolase (CBH), β-1,4-N-acetylglucosaminidase (NAG), leucine aminopeptidase (LAP), and acid phosphatase (ACP) were measured. In each quadrant, after removing the surface litter layer, a soil auger with a diameter of 5 cm was used to collect topsoil samples (0–10 cm and 10–20 cm soil layers) from three positions following the snake-like sampling method. The collected samples from each position were mixed and placed in self-sealing bags. For each sampling site, soils from the three quadrants were thoroughly mixed to form a composite sample. A total of 126 soil samples (21 sampling sites × 2 soil layers × 3 biological replicates) were obtained for laboratory analysis and were immediately transported to the laboratory using a cool box with ice packs. After removing plant residues and stones from the composite samples, the samples were sieved through a 2 mm sieve. In the laboratory, each mixed soil sample was divided into two parts: one part was stored at 4 °C for the determination of inorganic nitrogen (NO_3_^−^-N and NH_4_^+^-N), microbial biomass (MBC, MBN, MBP) and enzyme activities. Notably, the time interval from field sampling to laboratory storage was strictly controlled within 6 h to minimize the influence on enzymatic activity. The other part was air-dried for the determination of soil physicochemical properties.

### 2.3. Soil Analysis

#### 2.3.1. Soil Physicochemical Analysis

The soil pH was measured with a pH meter using an extract prepared at a soil-to-water ratio of 2.5:1. Soil organic carbon (SOC) content was determined using the potassium dichromate (K_2_Cr_2_O_7_) oxidation method [29]. For the determination of soil total nitrogen (TN) content, soil samples were digested with concentrated sulfuric acid (soil-to-solution ratio of 1:5) at 380 °C for 2 h, followed by analysis using a Kjeldahl nitrogen analyzer [30]. Soil nitrate nitrogen (NO_3_^−^-N) and ammonium nitrogen (NH_4_^+^-N) were extracted with a 2 mol/L potassium chloride (KCl) solution (soil-to-solution ratio of 1:5), and their contents were measured using a flow analyzer (Auto Analyzer; SEAL Analytical GmbH, Norderstedt, Germany). Soil total phosphorus (TP) content was determined using a spectrophotometer after digestion with a mixture of perchloric acid (HClO_4_) and sulfuric acid (H_2_SO_4_) [31]. The content of soil available phosphorus (SAP) was extracted using a 0.5 mol/L NaHCO_3_ solution (pH = 8.5) and determined by the molybdenum-antimony anti-spectrophotometric method [31]. Soil stoichiometric ratios (C:N, C:P, and N:P ratios) were calculated based on mass ratios (see Section 2.4).

#### 2.3.2. Analysis of Soil Microbial Biomass and Extracellular Enzyme Activity

Soil microbial biomass carbon (MBC), microbial biomass nitrogen (MBN), and microbial biomass phosphorus (MBP) were determined using the chloroform fumigation-extraction method [32]. Soil MBC, MBN, and MBP were calculated based on the differences in organic carbon and total nitrogen contents between fumigated and non-fumigated (control) samples. Extraction coefficients of 0.45, 0.54, and 0.40 [33] were used for the calculation of soil MBC, MBN, and MBP, respectively. For each sample, three replicates were set up, and the samples were fumigated with ethanol-free chloroform at 25 °C for 24 h. Subsequently, chloroform was removed by vacuum pumping. For the extraction of soil MBC and MBN, 50 mL of 0.5 mol/L potassium sulfate (K_2_SO_4_) solution was used to extract both the fumigated samples and control samples; for the extraction of soil MBP, 50 mL of 0.5 mol/L sodium bicarbonate (NaHCO_3_) solution was used. After extraction, the samples were shaken for 30 min at a speed of 170–180 r/min and then filtered through Whatman 42 filter paper. The extracts were stored frozen until analysis: MBC and MBN were determined using an Elemental Carbon-Nitrogen Analyzer (multN/C 2100s, analytik jena, Germany), while MBP was measured with a spectrophotometer.

Soil extracellular enzyme activities were measured using the 96-well microplate fluorometric method according to the protocol described by Saiya-Cork et al., (2002) [34]. The enzymes determined included two carbon-acquiring enzymes (β-1,4-glucosidase and β-1,4-cellobiohydrolase; abbreviated as BG and CBH, respectively), two nitrogen-acquiring enzymes (β-1,4-N-acetylglucosaminidase and leucine aminopeptidase; abbreviated as NAG and LAP, respectively), and one phosphorus-acquiring enzyme (acid phosphatase, ACP). The brief procedure was as follows: 0.02 g of soil sample was weighed into an Erlenmeyer flask, and 50 mL of 50 mM acetate buffer (pH = 5) was added. The mixture was stirred with a magnetic stirrer at 600 rpm for approximately 30 min. Subsequently, 70 μL of the supernatant was transferred to a 96-well microplate, and 130 μL of 200 mM substrate solution was added to each sample well. The substrates used were as follows: 4-methylumbelliferyl-β-D-glucopyranoside (MUB-β-D-glucoside) for BG, 4-methylumbelliferyl-β-D-cellobioside (MUB-β-D-cellobioside) for CBH, 4-methylumbelliferyl-N-acetyl-β-D-glucosaminide (MUB-N-acetyl-β-D-glucosaminide) for NAG, L-leucine-7-amido-4-methylcoumarin (L-leu-AMC) for LAP, and 4-methylumbelliferyl phosphate (MUB-phosphate) for ACP. The 96-well microplate was incubated at 20 °C for 4 h (for BG, CBH, NAG, and LAP) and 2 h (for ACP), respectively. After incubation, 10 μL of 1.0 M sodium hydroxide (NaOH) was added to each well to terminate the reaction. Immediately afterward, a fluorescence spectrophotometer (Tecan Spark, Zurich, Switzerland) was used to measure the fluorescence intensity. The excitation and emission wavelengths were set as follows: 400 nm for BG and NAG, 540 nm for CBH, 405 nm for LAP, and 660 nm for ACP. Extracellular enzymes and their functions, substrates, and incubation time are presented in Table 2.

### 2.4. Calculations

#### 2.4.1. Enzyme Stoichiometric Vector Analysis

In this study, the activities of carbon-acquiring enzymes (BG, CBH), nitrogen-acquiring enzymes (NAG, LAP), and phosphorus-acquiring enzymes (ACP) (μmol·d^−1^·g^−1^) were used to calculate the stoichiometric ratios of soil extracellular enzymes, with the formulas as follows:

E_*C:N*_ = Ln(*BG* + *CBH*)/Ln(*NAG* + *LAP*), (1)

E_*C:P*_ = Ln(*BG* + *CBH*)/Ln(*AP*), (2)

E_*N:P*_ = Ln(*NAG* + LAP)/Ln(*AP*). (3)

Nutrient limitation characteristics of soil microorganisms were quantified through vector analysis of soil enzyme stoichiometry, with the calculation formulas based on the theory proposed by Moorhead et al., (2016) [35]:

Length = SQRT(*x*^2^ + *y*^2^), (4)

Angle (degrees) = DEGREES(ATAN2(*x*; *y*)). (5)

In the formulas, higher vector length indicated relatively higher C versus nutrient acquisition strategies, and higher vector angle suggested higher P versus N acquisition efforts. Vector angle >45° means microbial relative P limitation and vector angle <45° means microbial relative N limitation [36]. Microbial relative P limitation increases, and microbial relative N limitation decreases with vector angle. Here, *x* denotes the relative activity of carbon and phosphorus acquiring enzymes, calculated as [(BG + CBH)/(BG + CBH + AP)]; y denotes the relative activity of carbon and nitrogen acquiring enzymes, calculated as [(BG + CBH)/(BG + CBH + NAG + LAP)].

#### 2.4.2. Stoichiometric Homeostasis

To evaluate the homeostasis (*H’*) response of soil microbial communities to carbon-to-nitrogen (C:N), carbon-to-phosphorus (C:P), and nitrogen-to-phosphorus (N:P) ratios across different altitudinal gradients, the following formula was adopted [37]:

*H*’ = 1/|*m*| (6)

Here, *m* represents the slope of the linear regression between the Ln of nutrient ratios and the natural logarithm of microbial biomass ratios: specifically, the slope between Ln(nutrient carbon-to-nitrogen ratio, Ln(N_C:N_)) and Ln(microbial biomass carbon-to-nitrogen ratio, Ln(B_C:N_)), the slope between Ln(nutrient carbon-to-phosphorus ratio, Ln(N_C:P_)) and Ln(microbial biomass carbon-to-phosphorus ratio, Ln(B_C:P_)), or the slope between Ln(nutrient nitrogen-to-phosphorus ratio, Ln(N_N:P_)) and Ln(microbial biomass nitrogen-to-phosphorus ratio, Ln(B_N:P_)).Strong homeostasis was indicated when the regression *p*-value < 0.05 and *H*’ > 1; weak or no homeostasis was indicated when the regression *p*-value < 0.05 and *H*’ ≈ 1; and strict homeostasis was indicated when the regression *p*-value > 0.05 [38].

### 2.5. Statistical Analysis

A combined map of the sampling site distribution and terrain was generated using ArcGIS 10.8.1 software. Data processing and preliminary analysis were performed with Microsoft Excel 2016, while statistical analyses were conducted using IBM SPSS Statistics (SPSS 27.0). One-way analysis of variance (One-way ANOVA) followed by the least significant difference (LSD) post hoc test was used to assess the statistical significance of differences in soil nutrients, extracellular enzyme activities, stoichiometric ratios, and microbial metabolic limitations (vector angle and vector length). Linear regression models were applied to determine the relationships between enzyme activities. Origin 2022 software was used for the following analyses and visualizations: analysis of microbial biomass and its stoichiometric ratios, standardized major axis (SMA) regression for enzyme activity stoichiometry, and plotting of Pearson correlation graphs. Principal component analysis (PCA) was performed using the *FactoMineR* and *factoextra* packages in RStudio 4.2.1 [39,40]; the *lavaan* package was used to construct structural equation models (SEM) [41]. All figures were plotted using the “ggplot2” package (Version 3.5.0) in RStudio 4.2.1.

## 3. Results

### 3.1. Soil Stoichiometric Ratios

#### 3.1.1. Soil Organic Carbon, Total Nitrogen, Total Phosphorus and Soil Available Nutrient

**Figure 2:**
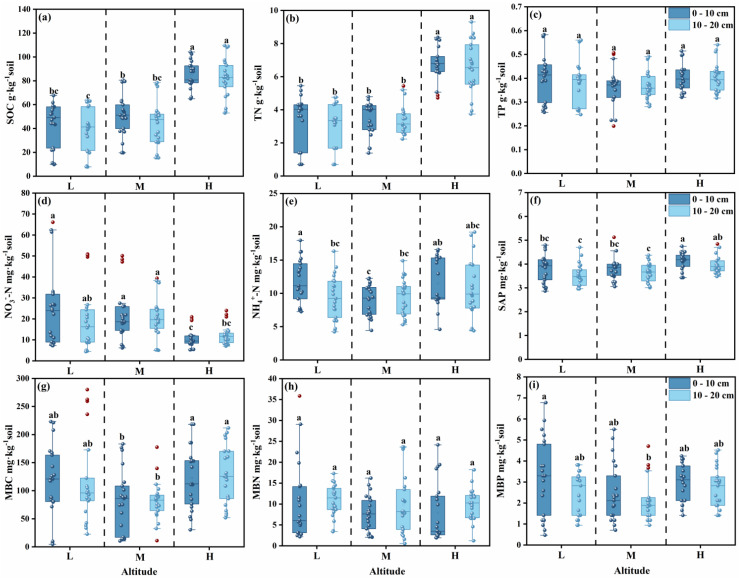
Soil total nutrients, available nutrients, and microbial biomass under different altitudinal gradients. SOC: Soil Organic Carbon. (**a**) TN: Total Nitrogen; (**b**) TP: Total Phosphorus; (**c**) NO_3_^−^-N: Nitrate Nitrogen; (**d**) NH_4_^+^-N: Ammonium Nitrogen; (**e**) SAP: Soil Available Phosphorus; (**f**) MBC: Microbial Biomass Carbon; (**g**) MBN: Microbial Biomass Nitrogen; (**h**) MBP: Microbial Biomass Phosphorus; (**i**) different letters indicate values significantly different from each other (*p* < 0.05) by one-way analysis of variance (*ANOVA*).

With the increase in altitude, the contents of soil organic carbon (SOC) and total nitrogen (TN) gradually increased. Compared with the low altitude (LA), the contents of SOC and TN in the 20 cm soil layer at the high altitude (HA) doubled, while the content of total phosphorus (TP) showed no significant change (Figure 2a–c). The nitrate nitrogen (NO_3_^−^-N) content at the middle altitude (MA) was significantly 84.65% higher than that at the high altitude, whereas the ammonium nitrogen (NH_4_^+^-N) content at the high altitude was 20.91% higher than that at the middle altitude; in addition, the soil available phosphorus (SAP) content at the high altitude was increased by 10.49% and 9.30% compared with that at the low altitude and middle altitude, respectively (Figure 2d–f). At the low altitude, the contents of total soil nutrients and available nutrients in the 0–10 cm soil layer were higher than those in the 10–20 cm soil layer.

#### 3.1.2. Stoichiometric Ratios

**Figure 3:**
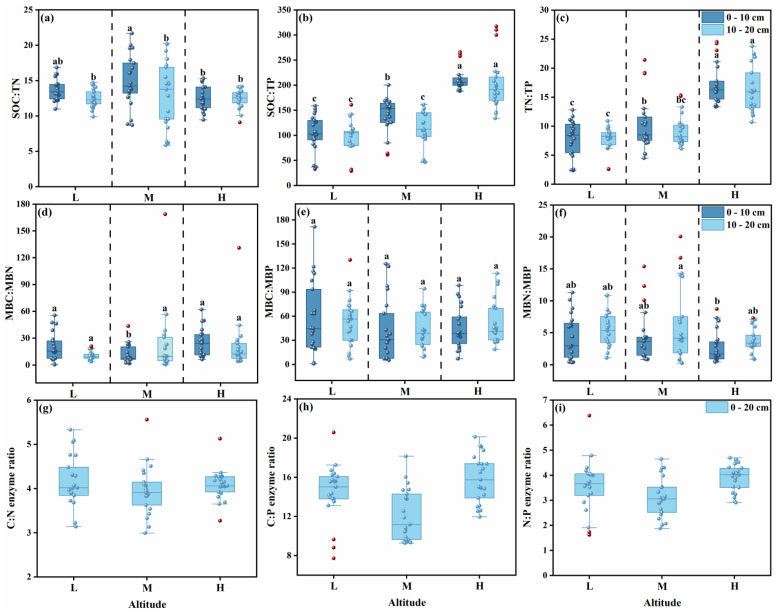
Soil nutrient stoichiometric ratios (SOC:TN (**a**), SOC:TP (**b**), TN:TP (**c**)), microbial biomass stoichiometric ratios (MBC:MBN (**d**), MBC:MBP (**e**), MBN:MBP (**f**)) and extracellular enzyme stoichiometric ratios (Enzyme C:N ratio (**g**), Enzyme C:P ratio (**h**), Enzyme C:N ratio (**i**)) in the 0~20 cm soil layer across different altitudinal gradients. In the figure, different letters indicate values significantly different from each other (*p* < 0.05) by one-way analysis of variance (*ANOVA*).

SOC:TN showed no significant change along the altitudinal gradient, while both SOC:TP and TN:TP gradually increased with rising altitude—with the ratios at high altitude being twice those at low altitude (Figure 3b,c). From the perspective of different soil layers, the soil nutrient stoichiometric ratios in the 0–10 cm layer were slightly higher than those in the 10–20 cm layer across all altitudinal gradients (Figure 3).

### 3.2. Microbial Biomass Stoichiometric Ratios

#### 3.2.1. Microbial Biomass

The contents of MBC and MBP were relatively low in the middle altitude region (M), while the content of MBN showed no significant change (Figure 2).

#### 3.2.2. Stoichiometric Ratios

There were no significant differences in the MBC:MBN and MBC:MBP (Figure 3d,e). However, the MBN:MBP was significantly higher in the altitudinal gradient of 3200–3500 masl; specifically, it was 44.3% higher in the 10–20 cm soil layer than in the 0–10 cm layer, and was the lowest in the 0–10 cm soil layer at high altitude (Figure 3f).

### 3.3. Characteristic of Extracellular Enzyme Activity Ratios

#### 3.3.1. Extracellular Enzyme Activities

**Figure 4:**
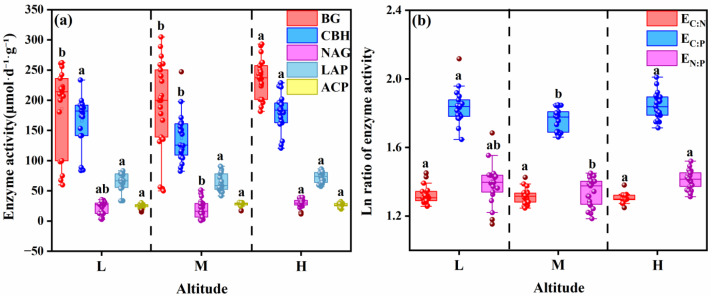
Soil Extracellular enzyme activities of each type and natural logarithmic ratios of enzyme activities between each pair of carbon (C)-, nitrogen (N)-, and phosphorus (P)-related enzymes under different altitudinal gradients. BG: β-1,4-Glucosidase; CBH: β-1,4-Cellobiohydrolase; NAG: β-1,4-N-Acetylglucosaminidase; LAP: Leucine Aminopeptidase; ACP: Acid Phosphatase; E_C:N_: Ln(BG + CBH)/Ln(NAG + LAP); E_C:P_: Ln(BG + CBH)/Ln(AP); E_N:P_: Ln(NAG + LAP)/Ln(AP). In the figure, different letters indicate values significantly different from each other (*p* < 0.05) by one-way analysis of variance (*ANOVA*).

Enzymes related to carbon and nitrogen cycling (BG, CBH, NAG, and LAP) all reached their maximum activities at the high altitudinal gradient; in particular, the activities of BG and NAG were significantly increased by 26.77% and 30.88%, respectively, compared with those at the low altitude, while the activities of LAP and ACP showed no significant change (Figure 4a).

#### 3.3.2. Enzyme Stoichiometric Ratios

Compared with the middle altitude, the enzyme C:P ratio (E_C:P_) and enzyme N:P ratio (E_N:P_) at the high altitude significantly increased by 30.46% and 24.11%, respectively (Figure 3h,i). The E_N:P_ was significantly higher at the high altitude, the E_C:P_ was significantly lower at the middle altitudinal gradient, and the enzyme C:N ratio (E_C:N_) showed no significant difference along the altitudinal gradient (Figure 4b).

### 3.4. Characteristics of Microbial Stoichiometric Homeostasis and Microbial Nutrient Limitation Under Different Altitudinal Gradients

**Figure 5:**
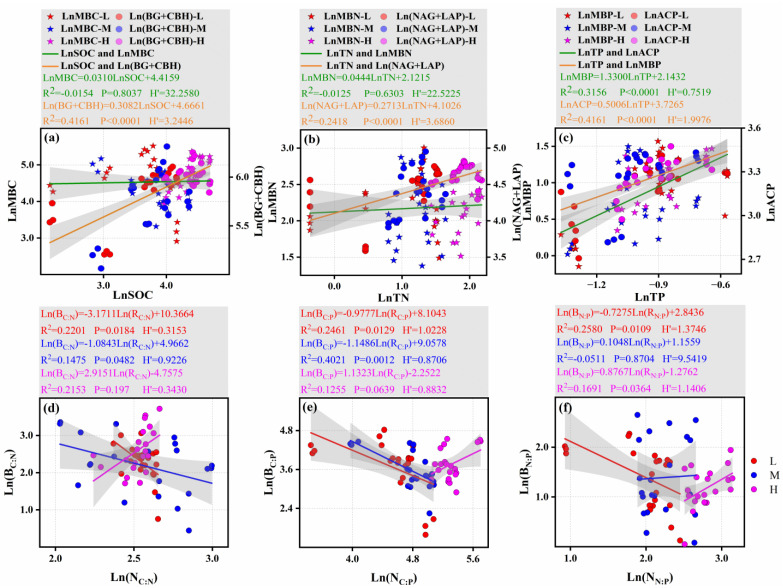
Ln-transformed stoichiometric relationship diagrams between soil nutrient elements, soil microbial biomass, and C-N-P-related enzymes, as well as between soil microbial biomass and its resources. The figure includes: relationships of carbon homeostasis (**a**), nitrogen homeostasis (**b**), and phosphorus homeostasis (**c**) across different altitudinal gradients; and relationships of carbon-nitrogen homeostasis (**d**), carbon-phosphorus homeostasis (**e**), and nitrogen-phosphorus homeostasis (**f**) across different altitudinal gradients. Abbreviations: R_C:N_, R_C:P_, and R_N:P_ represent the carbon-to-nitrogen ratio, carbon-to-phosphorus ratio, and nitrogen-to-phosphorus ratio of total soil nutrients, respectively; B_C:N_, B_C:P_, and B_N:P_ correspond to the carbon-to-nitrogen ratio, carbon-to-phosphorus ratio, and nitrogen-to-phosphorus ratio of soil microbial biomass, respectively.

As shown in Figure 4, along the altitudinal gradient, the logarithmic-transformed regression analysis between each soil nutrient element and carbon (C)-, nitrogen (N)-, and phosphorus (P)-related enzymes showed a significant positive correlation. The soil C and N elements showed good homeostasis (*H*’ far greater than 1). Notably, the homeostasis between soil C/N nutrients and C/N-related enzymes was stronger (*p* < 0.0001) (Figure 5a,b). In contrast, the *H*’ value between TP and MBP was less than 1 with *p* < 0.0001 (Figure 5c), indicating weak homeostasis; however, the homeostasis between TP and ACP was stronger (*H*’ > 1 and *p* < 0.0001) (Figure 5c). From the linear regression analysis between microbial biomass and soil nutrient stoichiometric ratios (Figure 5d–f), it can be observed that, at the low and middle altitudinal gradients, the LnB_C:N_ and LnB_C:P_ showed significant negative correlations with the LnN_C:N_ and LnN_C:P_, respectively; whereas, at the high altitudinal gradient, these relationships shifted to significant positive correlations. The LnB_N:P_ exhibited a weak positive correlation with the LnN_N:P_ at the middle and high altitudinal gradients, but a significant negative correlation at the low altitudinal gradient. In terms of homeostasis characteristics, across the three altitudinal gradients, the carbon-nitrogen (C-N) and carbon-phosphorus (C-P) elements exhibited relatively weak homeostasis (*H*′ < 1 or ≈ 1) (Figure 5d,e); while the nitrogen-phosphorus (N-P) elements showed strong homeostasis, which was particularly stronger at the low and high altitudinal gradients (Figure 5f).

**Figure 6:**
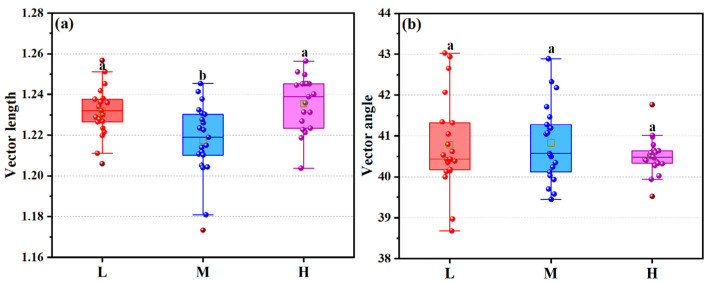
Relationship diagram between different altitudinal gradient treatments, vector length (**a**), and vector angle (**b**) derived from vector analysis of soil extracellular enzymes. In the figure, different letters indicate values significantly different from each other (*p* < 0.05) by one-way analysis of variance (*ANOVA*).

The vector length varied significantly (Figure 6a), compared with the middle altitude, the low and high altitudes showed stronger carbon limitation (1.2318 and 1.2355, *p* < 0.05). There was relative nitrogen limitation along the altitudinal gradient (Figure 6b), but the vector angle at high altitude reached the minimum value (40.5°), indicating relatively stronger nitrogen limitation.

### 3.5. Driving Mechanism of Soil Extracellular Enzymes on Soil Nutrients

**Figure 7:**
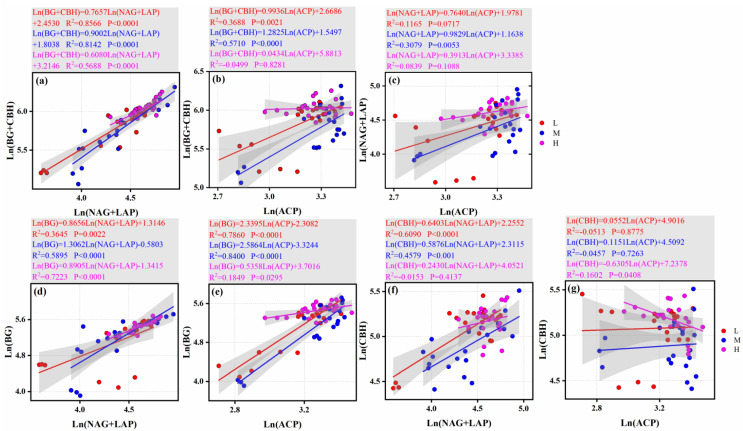
Results of standardized major axis (SMA) regression (Type II) analysis. The figure presents: (**a**) the relationship between carbon (C)-acquiring enzyme activity (represented by the sum of β-1,4-glucosidase (BG) and β-1,4-cellobiohydrolase (CBH)) and nitrogen (N)-acquiring enzyme activity (represented by the sum of β-1,4-N-acetylglucosaminidase (NAG) and leucine aminopeptidase (LAP)); (**b**) the relationship between C-acquiring enzyme activity (BG + CBH) and phosphorus (P)-acquiring enzyme activity (represented by acid phosphatase (ACP)); (**c**) the relationship between N-acquiring enzyme activity (NAG + LAP) and P-acquiring enzyme activity (ACP); (**d**) the relationship between β-1,4-glucosidase (BG) activity and nitrogen (N)-acquiring enzyme activity (represented by the sum of β-1,4-N-acetylglucosaminidase (NAG) and leucine aminopeptidase (LAP)); (**e**) the relationship between β-1,4-glucosidase (BG) activity and phosphorus (P)-acquiring enzyme activity (acid phosphatase (ACP)); (**f**) the relationship between β-1,4-cellobiohydrolase (CBH) activity and N-acquiring enzyme activity (represented by the sum of NAG and LAP); (**g**) the relationship between β-1,4-cellobiohydrolase (CBH) activity and P-acquiring enzyme activity (acid phosphatase (ACP)). All data were subjected to logarithmic transformation.

The carbon (C)-, nitrogen (N)-, and phosphorus (P)-related enzymes showed a certain degree of positive correlation across the three altitudinal gradients. Among them, the synergy between C- and N-cycling enzymes was the strongest across altitudes (with the highest R^2^) (Figure 7a). The pairwise correlations between C-, N-, and P-related enzymes were all significantly positive at the low and middle altitudinal gradients (*p* < 0.05 and relatively large slopes); in particular, the synergy between C- and N-cycling enzymes was even stronger (*p* < 0.0001 and relatively high R^2^). However, there was no significant correlation between C- and P-related enzymes, nor between N- and P-related enzymes at the high altitude (*p* > 0.05 and relatively low R^2^) (Figure 7a–c).

The correlations between key carbon cycling enzymes (BG, CBH) and nitrogen cycling enzymes ((NAG + LAP)) were generally significant (Figure 7d,f). However, the significance of the correlation between carbon cycling enzymes and phosphorus cycling enzymes (ACP) varied with altitude (Figure 7e,g); specifically, CBH and ACP showed a distinct negative correlation at high altitudes (Figure 7g). Compared with CBH, BG exhibited more significant correlations with nitrogen-cycling enzymes and phosphorus-cycling enzymes (with higher R^2^ and *p* < 0.05) (Figure 7).

### 3.6. Integrated Microbial Adaptation Mechanism of Altitudinal Gradient on Soil Nutrient Stoichiometry

**Figure 8:**
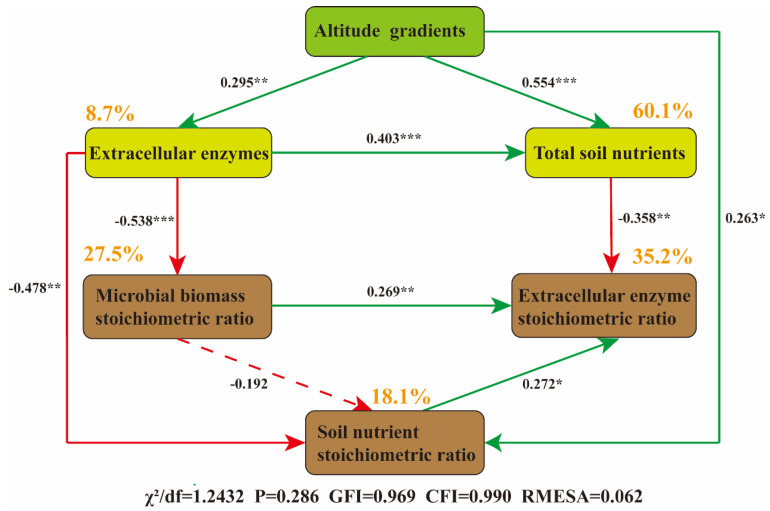
Results of the final piecewise structural equation model (SEM). The model illustrates the direct effects of the altitudinal gradient on soil nutrients and extracellular enzymes, as well as the indirect effects thereby induced among soil extracellular enzyme stoichiometry, microbial biomass stoichiometry, and soil nutrient stoichiometry. The path coefficients (correlation coefficients) beside the arrows were standardized using the mean values of each parameter. Green arrows and red arrows represent significant positive correlations and significant negative correlations, respectively (*p* < 0.05), while dashed lines indicate non-significant relationships (*p* > 0.05). The percentages labeled next to the variables represent the variance explained by the model (R^2^). Symbols *, **, and *** indicate statistical significance at the levels of *p* < 0.05, *p* < 0.01, and *p* < 0.001, respectively.

The final segmented structural equation model (*χ^2^*/*df* = 1.243, *p* = 0.286, *CFI* = 0.990, *RMSEA* = 0.062, indicating a good fit) showed that the altitudinal gradient significantly reduced microbial stoichiometric imbalance. Among the influencing factors, soil extracellular enzymes directly explained 8.7% of the total variation in microbial stoichiometric imbalance (Figure 8). The combined pathways of altitudinal gradient, soil extracellular enzymes, and microbial biomass stoichiometry explained 18.1% of the variation in soil nutrient stoichiometry (Figure 8). Notably, microbial biomass stoichiometry had no significant effect on soil nutrient stoichiometry (Figure 8). In addition, the piecewise SEM analysis revealed that the altitudinal gradient indirectly affected soil extracellular enzyme stoichiometry by altering soil extracellular enzymes, soil nutrients, and soil microbial biomass stoichiometry. Collectively, soil nutrients, soil nutrient stoichiometry and soil microbial biomass stoichiometry explained 35.2% of the total variation in soil extracellular enzyme stoichiometry (Figure 8).

## 4. Discussion

### 4.1. Soil Nutrients and Enzyme Activities Along the Altitudinal Gradient

With variation in the altitudinal gradient, soil nutrients and enzyme activities exhibited significant regular changes. With an increase in altitude, soil organic carbon (SOC) and total nitrogen (TN) showed an accumulation trend (Figure 2a,b), which is consistent with the research findings of Guan, Z.H., et al. [42]. This indicates that the low-temperature environment at high altitudes inhibits microbial decomposition activities, possibly hindering nutrient turnover and resulting in a large amount of nutrient retention [43]. In turn, microorganisms increase the secretion of carbon- and nitrogen-related enzymes (BG, CBH, NAG) to maintain a normal nutrient acquisition rate (Figure 4a). There is no significant change in phosphorus content (Figure 2c,f,i), which may be because phosphorus mainly originates from rock weathering. The differences in temperature, precipitation, etc., brought about by the altitudinal gradient have long-term and slow effects on rock weathering. Moreover, phosphorus has strong chemical stability in the soil, is not easily leached away with water, and is not easily rapidly mineralized or fixed by microorganisms [44]. Therefore, the phosphorus content remains relatively stable. Nitrate nitrogen is significantly higher at mid-altitudes (Figure 2d, *p* < 0.05) and lower at high altitudes, while ammonium nitrogen shows the opposite trend (Figure 2e, Figure S1a). This suggests that the basal metabolism of nitrifying bacteria may be relatively adapted to the temperature at mid-altitudes, and the absorption rate of nitrate nitrogen by mid-altitude vegetation (such as *Elymus dahuricus*) may be lower than the nitrification rate [45], leading to the gradual accumulation of nitrate nitrogen.

### 4.2. Microbial Nutrient Limitation Along the Altitudinal Gradient

Changes in the altitudinal gradient will lead to changes in soil nutrients. The nutrient status affects microbial activity and the utilization strategy of soil organic matter by microorganisms [46,47,48,49], ultimately altering the microbial metabolic processes in alpine meadow ecosystems [50]. Soil conditions and microbial demands may be influenced by soil available carbon, nutrients, microclimate, and chemical properties [51,52]. The soil pH at high altitudes ranges from weakly acidic to neutral (6.12–7.45), and low temperatures will cause microorganisms to reduce the decomposition rate of soil organic matter (SOM). Compared with mid and low altitudes, the activities of carbon and nitrogen-related enzymes are higher at high altitudes (Figure 4a), resulting in a carbon supply that cannot meet the demands of microorganisms and plants, thereby forming a relatively strong carbon limitation. Another reason may be that environmental conditions such as low temperature and precipitation at high altitudes will change the vegetation composition (such as *Anemone vitifolia*, *Potentilla fruticosa*, etc.) (Table 1), leading to an increase in the lignin and cellulose content of plant litter and a decrease in carbon substrate quality [53,54]. Such substrates are difficult to be rapidly decomposed by microorganisms, enhancing carbon limitation. In addition, this study found that the relative nitrogen limitation is stronger at high altitudes, which differs from the findings of Dandan Zhang et al. [55], who reported that extreme environmental conditions and lower pH at high altitudes reduce enzyme activity, possibly exacerbating relative phosphorus limitation. Probably because temperature is a key driving factor regulating soil nitrogen cycling [56]. The low-temperature environment will significantly inhibit microbial activity, slow down the mineralization process of soil organic nitrogen, and simultaneously reduce the efficiency of nitrogen transformations such as nitrification and denitrification. Although the TN content in high-altitude soils is relatively high (Figure 2b), low temperatures result in an available nitrogen (Figure 2d) supply rate that is much lower than the demands of plants and microorganisms [57], forming a pattern of relative nitrogen limitation.

### 4.3. Regulation Mechanism of Soil Nutrients on Extracellular Enzyme Activities from the Perspective of Microbial Adaptation Mechanisms

Due to the distribution of microorganisms being affected by multiple environmental factors along the altitudinal gradient, there is a differential regulation of extracellular enzyme activities [58]. The low-altitude environment has relatively higher temperatures and abundant nutrients, supporting the survival of a highly diverse microbial community [42], enhancing enzyme activities and promoting rapid carbon and nitrogen turnover. The increase in microbial biomass carbon (MBC) at high altitudes may be related to adaptive changes in the microbial community structure. Along the altitudinal gradient, the dominance of fungi over bacteria gradually increases [59,60]. The dominance of meadow fungi increases with increasing humidity, decreasing temperature, and decreasing pH. Studies have shown that the abundance of genes encoding bacterial decomposing enzymes increases with altitude [61], and the microbial taxa secreting extracellular enzymes may vary along the altitudinal gradient. By adjusting the community composition and optimizing enzymatic reactions, microorganisms maintain the balance of carbon, nitrogen, and phosphorus metabolism (Figure 7a–c). In addition, studies have shown that the interaction between plants and microorganisms also plays an important regulatory role along the altitudinal gradient [62]. Root exudates are abundant in low-altitude plants, stimulating microorganisms to secrete extracellular enzymes; the quality of plant litter changes at high altitudes, prompting microorganisms to change their enzyme investment strategies [63], synergistically regulating extracellular enzyme activities (Figure 8).

Changes in the altitudinal gradient may alter the microbial secretion strategy of extracellular enzymes, thereby affecting the microbial biomass stoichiometric ratio (Figure S1). The microbial biomass stoichiometric ratio explains 35.2% of the variation in soil extracellular enzyme stoichiometric ratio (Figure 8). Both microbial biomass stoichiometry and nutrient stoichiometry are negatively affected by extracellular enzymes. The reason may be that when enzyme activity is high, microorganisms can acquire a large amount of nutrients more efficiently and do not need to maintain a high proportion of their own biomass to store nutrients [64], resulting in a decrease in their stoichiometric ratio (Figure 8). The low carbon and nitrogen contents at mid-altitudes (Figure 2a,b) will increase the microbial demand for phosphorus-acquiring enzymes (Figure 4a), leading to a decrease in the enzyme carbon-phosphorus ratio and nitrogen-phosphorus ratio (Figure 3h,i), which is consistent with the theory that “enzyme stoichiometry is reversely adjusted with substrate nutrient availability” [65]. At low altitudes, the relatively higher temperature may drive the acceleration of carbon and nitrogen mineralization rates, and microbial metabolism is balanced, with the activities of carbon, nitrogen, and phosphorus-acquiring enzymes being coordinately regulated [57], resulting in no significant differences in enzyme ratios (Figure 3g–i). This pattern is consistent with the research conclusions of multi-regional altitudinal gradient studies such as those focused on Maxian Mountain, Qilian Mountain, and Medog. In summary, the change in soil extracellular enzyme activity is less affected by nutrient limitation caused by altitude, indicating that there is a strong homeostasis characteristic among microbial stoichiometries (Figure 5). Microorganisms maintain the C, N, and P balance in the soil system by adjusting their own biomass stoichiometric ratio and enzyme secretion strategy [66], reflecting the adaptive characteristics of carbon, nitrogen, and phosphorus enzyme activity ratios to the nutrient supply pattern and microbial metabolic strategy in high-altitude environments.

### 4.4. Association of Research Findings with Global Change, Research Limitations, and Future Directions

Under the background of global climate change, the increase in grassland temperature may change the current temperature pattern along the altitudinal gradient, affecting soil microbial communities, enzyme activities, and nutrient cycling [67]. High-altitude microorganisms may enhance metabolic activity due to temperature increase, changing nutrient storage patterns; changes in precipitation patterns may also disrupt the current regulation of soil processes by water [68]. In studies on carbon enzymes in alpine meadows, it is recommended to select BG (*p* < 0.05) instead of CBH (Figure 7f,g). In addition, this study has certain limitations: it failed to determine soil physical and chemical properties such as soil texture and soil mechanical composition, only focused on a specific meadow altitudinal gradient, and lacked cross-regional comparisons; furthermore, the regulatory mechanisms of microbial communities and enzyme activities were not explored at the molecular level. Future research can be expanded to meadows in different geographical regions, conducting long-term multi-factor control experiments; combining technologies such as metagenomics and transcriptomics to analyze the molecular mechanisms of microbial regulation of soil processes [69], providing a more solid foundation for predicting the response of soil ecosystems to global change.

## 5. Conclusions

In conclusion, we found that with increasing altitude, total soil nutrients gradually increase and are mostly accumulated in the topsoil. Soil extracellular enzymes link organic matter decomposition and microbial metabolism, and their activities and ratios further regulate the stoichiometric balance between microorganisms and soil. As an initial driving factor, altitude directly affects the accumulation of total soil nutrients; mainly through climatic conditions. The climatic conditions and inefficient available nutrients in high-altitude areas lead to an increase in microbial energy demand, thereby exacerbating the relative carbon limitation. Changes in environmental conditions induce alterations in the structures of vegetation and microbial communities, which may affect the rate of organic matter decomposition by microorganisms and the secretion strategy of extracellular enzymes. Microorganisms maintain relatively stable characteristics to meet their own metabolic needs. It is highly likely that changes in the microenvironment, rather than soil nutrient status, are the key factors driving microbial metabolic limitation and nutrient use efficiency. However, this study still has room for improvement, and additional factors such as soil granulometric composition need to be considered. Overall, our study reveals the altitudinal variation in soil microbial nutrient limitation in meadow ecosystems, providing a basis for understanding nutrient cycling in meadow ecosystems under environmental changes.

## Figures and Tables

**Table 1:** Sample information of alpine meadows at different altitudinal gradients in the northeaster Qinghai–Tibet Plateau.

Sample Number	Dominant Species	Altitude/masl	Longitude and Latitude
S1	*Festuca ovina*	3749	102°7′40″ E, 34°15′5″ N
S2	*Elymus dahuricus*	3453	102°15′35″ E, 34°21′4″ N
S3	*Elymus dahuricus*	3122	102°25′57″ E, 34°37′23″ N
S4	*Elymus dahuricus*, *Anemone vitifolia*	3646	102°5′50″ E, 34°13′59″ N
S5	*Elymus dahuricus*	3459	102°14′16″ E, 34°25′54″ N
S6	*Elymus dahuricus*	3081	102°39′54″ E, 34°32′32″ N
S7	*Hippophae rhamnoides*, *Festuca ovina*, *Elymus dahuricus*	3692	102°10′50″ E, 34°15′55″ N
S8	*Festuca ovina*	3413	102°15′37″ E, 34°31′47″ N
S9	*Festuca ovina*	3139	102°28′25″ E, 34°36′24″ N
S10	*Elymus dahuricus*	3762	102°1′46″ E, 34°13′32″ N
S11	*Elymus dahuricus*	3436	102°18′1″ E, 34°35′5″ N
S12	*Leontopodium nanum*	3092	102°32′25″ E, 34°34′11″ N
S13	*Anemone vitifolia*	3723	102°12′30″ E, 34°17′57″ N
S14	*Potentilla fruticosa*	3389	102°22′7″ E, 34°44′26″ N
S15	*Taraxacum mongolicum*, *Lycium barbarum*, *Hippophae rhamnoides*	2996	102°36′18″ E, 34°32′38″ N
S16	*Potentilla fruticosa*	3722	102°13′26″ E, 34°19′44″ N
S17	*Elymus dahuricus*	3316	102°19′55″ E, 34°37′56″ N
S18	*Juniperus formosana*	3128	102°23′54″ E, 34°37′5″ N
S19	*Lycium barbarum*	3759	102°9′34″ E, 34°15′ N
S20	*Hippophae rhamnoides*, *Gentiana scabra*	3317	102°21′40″ E, 34°41′15″ N
S21	*Rhodiola crenulata*, *Lycium barbarum*	3141	102°30′58″ E, 34°35′30″ N

**Table 2:** Extracellular enzymes and their functions, substrates, and incubation time.

Enzyme	Abbreviation	Function	Substrate	Time (h)
β-1,4-glucosidase	BG	EC3.2.1.21	ρNP-β-D-glucopyranoside	4
β-1,4-cellobiohydrolase	CBH	EC3.2.1.91	4-ρNP-β-D-cellobioside	4
β-1,4-N-acetylglucosaminidase	NAG	EC3.2.1.14	ρNP-N-acetyl-β-D-glucosaminide	4
Leucine aminopeptidase	LAP	EC 3.4.11.1	Leucine-ρ-nitroanilide	4
acid phosphatase	ACP	EC 3.1.3.1	ρNP-phosphate	2

## Data Availability

The original contributions presented in this study are included in the article/Supplementary Material. Further inquiries can be directed to the corresponding author.

## Supplementary Materials

The following supporting information can be downloaded at: https://www.mdpi.com/article/10.3390/microorganisms13122692/s1. Figure S1. Principal component analysis (PCA) results across different altitudinal gradients. (a) PCA of total soil nutrients (soil organic carbon (SOC), total nitrogen (TN), total phosphorus (TP)), available nutrients (nitrate nitrogen (NO_3_^−^-N), ammonium nitrogen (NH_4_^+^-N), soil available phosphorus (SAP)), microbial biomass carbon, nitrogen, and phosphorus (MBC, MBN, MBP), and activities of carbon (C)-, nitrogen (N)-, and phosphorus (P)-related enzymes (β-1,4-glucosidase (BG), β-1,4-N-acetylglucosaminidase (NAG), β-1,4-cellobiohydrolase (CBH), leucine aminopeptidase (LAP), and acid phosphatase (ACP)); (b) PCA of total soil nutrient stoichiometric ratios (carbon-to-nitrogen ratio (N_C:N_), carbon-to-phosphorus ratio (N_C:P_), nitrogen-to-phosphorus ratio (N_N:P_), carbon-to-nitrogen-to-phosphorus ratio (N_C:N:P_)), microbial biomass C-N-P stoichiometric ratios (carbon-to-nitrogen ratio (M_C:N_), carbon-to-phosphorus ratio (M_C:P_), nitrogen-to-phosphorus ratio (M_N:P_), carbon-to-nitrogen-to-phosphorus ratio (M_C:N:P_)), and stoichiometric ratios of C-N-P-related enzyme activities (β-1,4-glucosidase (BG), β-1,4-N-acetylglucosaminidase (NAG), β-1,4-cellobiohydrolase (CBH), leucine aminopeptidase (LAP), and acid phosphatase (ACP)) (carbon-to-nitrogen ratio (E_C:N_), carbon-to-phosphorus ratio (E_C:P_), nitrogen-to-phosphorus ratio (E_N:P_), carbon-to-nitrogen-to-phosphorus ratio (E_C:N:P_)). Data S1: Soil pH Data of Sampling Sites.

## References

[1] Bocharnikov M.V. (2023). Climate-Related Gradients on Vegetation Diversity of The Altai-Sayan Orobiome (Southern Siberia). Geogr. Environ. Sustain..

[2] Tegegn M.G., Berlie A.B., Utallo A.U. (2024). Spatiotemporal variability and trends of intra-seasonal rainfall and temperature in the drought-prone districts of Northwestern Ethiopia. Discov. Sustain..

[3] Wu G.-L., Liu Y., Wang D., Zhao J. (2024). Divergent successions to shrubs- and forbs-dominated meadows decrease ecosystem multifunctionality of hillside alpine meadow. Catena.

[4] A’Bear A.D., Boddy L., Kandeler E., Ruess L., Jones T.H. (2014). Effects of isopod population density on woodland decomposer microbial community function. Soil Biol. Biochem..

[5] Peng S., Liu W., Xu G., Pei X., Millerick K., Duan B. (2021). A meta-analysis of soil microbial and physicochemical properties following native forest conversion. Catena.

[6] Acosta B., Sánchez-Jardón L., del Pozo A., García-Ibáñez E., Casado M.A., Montalvo J., Pineda F.D. (2008). Grassland species composition and morpho-functional traits along an altitudinal gradient in a Mediterranean environment: Relationship with soil water availability and evaporative dynamic. Acta Oecol..

[7] Chen Y., Zhang Y., Bai E., Piao S., Chen N., Zhao G., Zheng Z., Zhu Y. (2022). The stimulatory effect of elevated CO_2_ on soil respiration is unaffected by N addition. Sci. Total Environ..

[8] Dolker T., Mukherjee A., Agrawal S.B., Agrawal M. (2020). Responses of a semi-natural grassland community of tropical region to elevated ozone: An assessment of soil dynamics and biomass accumulation. Sci. Total Environ..

[9] Du H., Pan J., Zhang C., Yang X., Wang C., Lin X., Li M. (2023). Analogous assembly mechanisms and functional guilds govern prokaryotic communities in mangrove ecosystems of China and South America. Microbiol. Spectr..

[10] Zheng X., Lin C., Guo B., Yu J., Ding H., Peng S., Sveen T.R., Zhang Y. (2020). Effects of re-vegetation restoration on soil bacterial community structure in degraded land in subtropical China. Eur. J. Soil Biol..

[11] Ren C., Zhou Z., Guo Y., Yang G., Zhao F., Wei G., Han X., Feng L., Feng Y., Ren G. (2021). Contrasting patterns of microbial community and enzyme activity between rhizosphere and bulk soil along an elevation gradient. Catena.

[12] Xing J., Chen W., Song C., Li X., Wang Q., Song Y. (2025). N deposition affects litter decomposition in evergreen broad-leaf forests by reducing soil enzyme activities and altering the structure of microbial communities. Plant Soil.

[13] Wang S., Liu Y., Chen L., Yang H., Wang G., Wang C., Dong X. (2021). Effects of excessive nitrogen on nitrogen uptake and transformation in the wetland soils of Liaohe estuary, northeast China. Sci. Total Environ..

[14] Yang S., Xu Z., Wang R., Zhang Y., Yao F., Zhang Y., Turco R.F., Jiang Y., Zou H., Li H. (2017). Variations in soil microbial community composition and enzymatic activities in response to increased N deposition and precipitation in Inner Mongolian grassland. Appl. Soil Ecol..

[15] Xu Z., Hu Z., Jiang L., Qiu T., Li W., Anthony M.A. (2025). Contrasting wood carbon quality of angiosperms and gymnosperms drives fungal-mediated decomposition responses to nutrient enrichment. For. Ecol. Manag..

[16] Hu M., Yan R., Wu H., Ni R., Zhang D., Zou S. (2023). Linking soil phosphorus availability and phosphatase functional genes to coastal marsh erosion: Implications for nutrient cycling and wetland restoration. Sci. Total Environ..

[17] Saleem M., Pervaiz Z.H., Traw M.B. (2015). Theories, Mechanisms and Patterns of Microbiome Species Coexistence in an Era of Climate Change. Microbiome Community Ecology.

[18] King A.E., Hofmockel K.S. (2017). Diversified cropping systems support greater microbial cycling and retention of carbon and nitrogen. Agric. Ecosyst. Environ..

[19] Drahorad S., Felix-Henningsen P., Siemens J., Marschner B., Heinze S. (2022). Patterns of enzyme activities and nutrient availability within biocrusts under increasing aridity in Negev desert. Ecosphere.

[20] Johnson C., Delattre H., Hayes C., Soyer O.S. (2021). An Evolutionary Systems Biology View on Metabolic System Structure and Dynamics. Evolutionary Systems Biology.

[21] Wang W., Wang W., Yu S., Zhang H., Yang J., Li X. (2024). Structural Characteristics and Driving Factors of Rhizosphere Microbial Communities in the Rhizosphere of Six Stipa Species Across the Ningxia Steppe. Sustainability.

[22] Yang H.X.L. (2025). Retention of fragmented fine woody debris enhances soil health, microbial activity, and ecosystem functions in urban forests of Northeast China. Plant Soil.

[23] Yao F., Qi W., Cao Y., Liang J., Liu X., Liu Z., Lv Y., Wei W., Xu W., Yu Y. (2025). The effects of a combination of maize/peanut intercropping and residue return on soil microbial nutrient limitation in maize fields. Appl. Soil Ecol..

[24] Sui X., Bao X., Xie H., Ba X., Yu Y., Yang Y., He H., Liang C., Zhang X. (2025). Contrasting seasonal effects of legume and grass cover crops as living mulch on the soil microbial community and nutrient metabolic limitations. Agric. Ecosyst. Environ..

[25] Smith A.P., Marín-Spiotta E., Balser T. (2015). Successional and seasonal variations in soil and litter microbial community structure and function during tropical postagricultural forest regeneration: A multiyear study. Glob. Change Biol..

[26] Jiao P.-Y., Guo W., Chen Z.-L., Liu X., Hu Y.-L., Wang Y.-Z. (2022). Soil Enzyme Stoichiometric Characteristics of Pinus massonianaPlantations at Different Stand Ages in Mid-subtropical Areas. Huan Jing Ke Xue Huanjing Kexue.

[27] Zhou C., Dun X., Tang Q., Long Y., Du J., Bao M. (2025). Elevation-dependent shifts in soil phosphorus forms and phosphorus-solubilizing microbial diversity suggest enhanced bioavailable phosphorus cycling with rising temperatures. Microbiol. Spectr..

[28] Bai J., Sturchio M.A., Luo Y., Lin G., Cheng X. (2025). Species turnover and climates co-dominate the carbon–water relationship in grasslands along an elevational gradient. Funct. Ecol..

[29] Walkley A., Black I.A. (1934). An examination of the degtjareff method for determining soil organic matter, and a proposed modification of the chromic acid titration method. Soil Sci..

[30] Bremner J., Mulvaney C. (1982). Nitrogen—Total. Methods of Soil Analysis, Part 2, Chemical and Microbiological Properties.

[31] Olsen S.R., Sommers L.E., Page A.L., Miller R.H., Keeney D.R. (1982). Phosphorous. Methods of Soil Analysis, Part 2, Chemical and Microbiological Properties.

[32] Vance E.D., Brookes P.C., Jenkinson D.S. (1987). An extraction method for measuring soil microbial biomass C. Soil Biol. Biochem..

[33] Jenkinson D. (2004). Measuring soil microbial biomass. Soil Biol. Biochem..

[34] Saiya-Cork K.R., Sinsabaugh R.L., Zak D.R. (2002). The effects of long term nitrogen deposition on extracellular enzyme activity in an Acer saccharum forest soil. Soil Biol. Biochem..

[35] Moorhead D.L., Sinsabaugh R.L., Hill B.H., Weintraub M.N. (2016). Vector analysis of ecoenzyme activities reveal constraints on coupled C, N and P dynamics. Soil Biol. Biochem..

[36] Cui Y., Wang X., Zhang X., Ju W., Duan C., Guo X., Wang Y., Fang L. (2020). Soil moisture mediates microbial carbon and phosphorus metabolism during vegetation succession in a semiarid region. Soil Biol. Biochem..

[37] Luo H., Yu J., Li R., Gu J.-D., Luo L., Zhang Y., He Y., Xiao Y., Deng S., Zhang Y. (2022). Microbial biomass C:N:P as a better indicator than soil and ecoenzymatic C:N:P for microbial nutrient limitation and C dynamics in Zoige Plateau peatland soils. Int. Biodeterior. Biodegrad..

[38] Wang X., Li X., Wang Z., Long A., Ji X., Gong X., Jiang Y., Qi H. (2025). Straw return increased maize phosphorus uptake and grain yield by alleviating rhizosphere soil microbial metabolism limitation: Insights from ecoenzymatic stoichiometry. Plant Soil.

[39] Veen G.F., Olff H., Duyts H., Van Der Putten W.H. (2010). Vertebrate herbivores influence soil nematodes by modifying plant communities. Ecology.

[40] Njiru L.G., Yegon J.R., Mwithiga G., Micheni A., Gitari N.J., Mairura F.S. (2023). Restoring soil nutrient stocks using local inputs, tillage and sorghum-green gram intercropping strategies for drylands in Eastern Kenya. Heliyon.

[41] Lefcheck J.S., Freckleton R. (2015). piecewiseSEM: Piecewise structural equation modelling inr for ecology, evolution, and systematics. Methods Ecol. Evol..

[42] Guan Z.-H., Jia B., Niu Z.-q., Mou X.-M., Chen J., Li F.-C., Wu Y.-N., Ning S., Yakov K., Li X.G. (2025). Humidity controls soil organic carbon accrual in grassland on the Qinghai–Tibet Plateau. Soil Biol. Biochem..

[43] Cui H., Chen S., Song H., Liu Z., Chen J., Zhang A., Xiao S., Jiang X., Yang Z., Li X. (2024). Contrasting mechanisms of non-vascular and vascular plants on spatial turnover in multifunctionality in the Antarctic continent. J. Ecol..

[44] Bicharanloo B., Bagheri Shirvan M., Dijkstra F.A. (2022). Decoupled cycling of carbon, nitrogen, and phosphorus in a grassland soil along a hillslope mediated by clay and soil moisture. Catena.

[45] Nielsen P.L., Andresen L.C., Michelsen A., Schmidt I.K., Kongstad J. (2009). Seasonal variations and effects of nutrient applications on N and P and microbial biomass under two temperate heathland plants. Appl. Soil Ecol..

[46] Li J., Liu Y., Hai X., Shangguan Z., Deng L. (2019). Dynamics of soil microbial C:N:P stoichiometry and its driving mechanisms following natural vegetation restoration after farmland abandonment. Sci. Total Environ..

[47] Xiao W., Chen X., Jing X., Zhu B. (2018). A meta-analysis of soil extracellular enzyme activities in response to global change. Soil Biol. Biochem..

[48] Zhou L., Liu S., Shen H., Zhao M., Xu L., Xing A., Fang J., Sayer E. (2020). Soil extracellular enzyme activity and stoichiometry in China’s forests. Funct. Ecol..

[49] Zuccarini P., Asensio D., Ogaya R., Sardans J., Peñuelas J. (2020). Effects of seasonal and decadal warming on soil enzymatic activity in a P-deficient Mediterranean shrubland. Glob. Change Biol..

[50] Malik A.A., Martiny J.B.H., Brodie E.L., Martiny A.C., Treseder K.K., Allison S.D. (2020). Defining trait-based microbial strategies with consequences for soil carbon cycling under climate change. ISME J..

[51] Yang T., Li X., Hu B., Li F., Wei D., Wang Z., Huang L., Bao W. (2024). Climate and soil properties shape latitudinal patterns of soil extracellular enzyme activity and stoichiometry: Evidence from Southwest China. Appl. Soil Ecol..

[52] Wang C., Zhang R., Vilonen L., Qu Y., Fu X., Shi B., Cui H., Gao W., Cai H., Sun W. (2021). Grazing and nitrogen addition restructure the spatial heterogeneity of soil microbial community structure and enzymatic activities. Funct. Ecol..

[53] Thomas F.M., Molitor F., Werner W. (2013). Lignin and cellulose concentrations in roots of Douglas fir and European beech of different diameter classes and soil depths. Trees.

[54] Tan B., Yin R., Yang W., Zhang J., Xu Z., Liu Y., He S., Zhou W., Zhang L., Li H. (2020). Soil fauna show different degradation patterns of lignin and cellulose along an elevational gradient. Appl. Soil Ecol..

[55] Zhang D., Wu B., Li J., Cheng X. (2023). Soil microbial relative resource limitation exhibited contrasting seasonal patterns along an elevational gradient in Yulong Snow Mountain. Funct. Ecol..

[56] Zhang Z., Liu Y., Zhao W., Liu K., Chen Y., Wang F., Mao G., Ji M. (2025). Distinct genes and microbial communities involved in nitrogen cycling between monsoon- and westerlies-dominated Tibetan glaciers. Nat. Commun..

[57] Lie Z., Huang W., Liu X., Zhou G., Yan J., Li Y., Huang C., Wu T., Fang X., Zhao M. (2021). Warming leads to more closed nitrogen cycling in nitrogen-rich tropical forests. Glob. Change Biol..

[58] Yuan X., Niu D., Gherardi L.A., Liu Y., Wang Y., Elser J.J., Fu H. (2019). Linkages of stoichiometric imbalances to soil microbial respiration with increasing nitrogen addition: Evidence from a long-term grassland experiment. Soil Biol. Biochem..

[59] Chen Y.L., Ding J.Z., Peng Y.F., Li F., Yang G.B., Liu L., Qin S.Q., Fang K., Yang Y.H. (2016). Patterns and drivers of soil microbial communities in Tibetan alpine and global terrestrial ecosystems. J. Biogeogr..

[60] Zhang C., Lei S., Wu H., Liao L., Wang X., Zhang L., Liu G., Wang G., Fang L., Song Z. (2024). Simplified microbial network reduced microbial structure stability and soil functionality in alpine grassland along a natural aridity gradient. Soil Biol. Biochem..

[61] Chen L., Wang J., He L., Xu X., Wang J., Ren C., Guo Y., Zhao F. (2023). Metagenomic highlight contrasting elevational pattern of bacteria- and fungi-derived compound decompositions in forest soils. Plant Soil.

[62] Sun T., Wang Y., Guo Y., Jing X., Feng W. (2023). Contrasting elevational patterns of microbial carbon and nutrient limitation in soil from alpine meadow to desert. Catena.

[63] Ramin K.I., Allison S.D. (2019). Bacterial Tradeoffs in Growth Rate and Extracellular Enzymes. Front. Microbiol..

[64] Cleveland C.C., Liptzin D. (2007). C:N:P stoichiometry in soil: Is there a “Redfield ratio” for the microbial biomass?. Biogeochemistry.

[65] Sinsabaugh R.L., Follstad Shah J.J. (2010). Ecoenzymatic stoichiometry of recalcitrant organic matter decomposition: The growth rate hypothesis in reverse. Biogeochemistry.

[66] Sinsabaugh R.L., Lauber C.L., Weintraub M.N., Ahmed B., Allison S.D., Crenshaw C., Contosta A.R., Cusack D., Frey S., Gallo M.E. (2008). Stoichiometry of soil enzyme activity at global scale. Ecol. Lett..

[67] Dong L., Zeng W., Wang A., Tang J., Yao X., Wang W. (2020). Response of Soil Respiration and Its Components to Warming and Dominant Species Removal along an Elevation Gradient in Alpine Meadow of the Qinghai–Tibetan Plateau. Environ. Sci. Technol..

[68] Liu S., Ru J., Guo X., Gao Q., Deng S., Lei J., Jian S., Changchun Z., Shiqiang W., Yang Y. (2025). Altered precipitation and nighttime warming reshape the vertical distribution of soil microbial communities. Msystems.

[69] Sieradzki E.T., Nuccio E.E., Pett-Ridge J., Firestone M.K. (2023). Expression of macromolecular organic nitrogen degrading enzymes identifies potential mediators of soil organic N availability to an annual grass. ISME J..

