# Supplementary material for: Stoichiometric Responses of Soil Microbes and Enzymes to Altitudinal Gradients in Alpine Meadows
Source: Microorganisms. 2025 Nov 25;13(12):2692. doi: 10.3390/microorganisms13122692 (PMC12735218; doi:10.3390/microorganisms13122692)
Supplement: Supplementary file 1 [file microorganisms-13-02692-s001.zip › microorganisms-3952544-supplementary/Supplementary Materials Figure S1.pdf]

Appendix1 Supplementary Materials

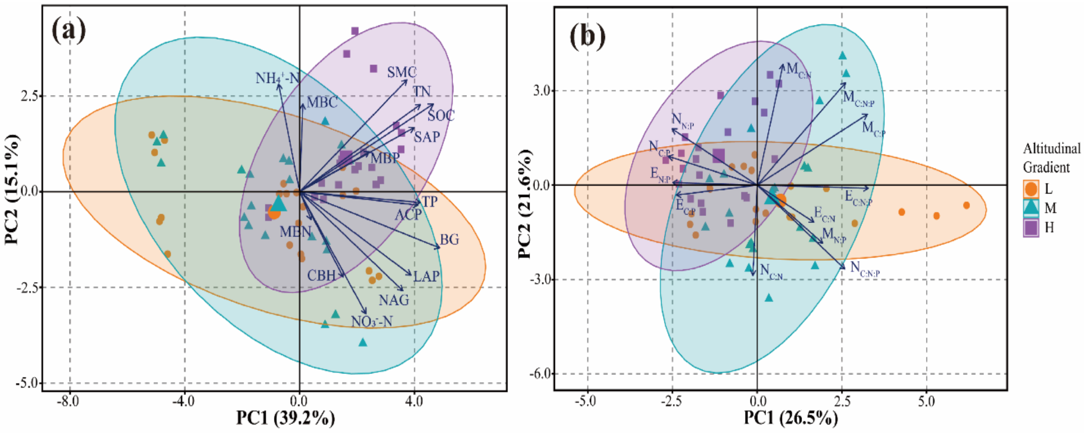

Figure.S1 Principal component analysis (PCA) results across different altitudinal gradients.(a) PCA of total soil nutrients (soil organic carbon (SOC), total nitrogen (TN), total phosphorus (TP)), available nutrients (nitrate nitrogen (NO_3^--N), ammonium nitrogen (NH_4^+-N), soil available phosphorus (SAP)), microbial biomass carbon, nitrogen, and phosphorus (MBC, MBN, MBP), and activities of carbon (C)-, nitrogen (N)-, and phosphorus (P)-related enzymes (β-1,4-glucosidase (BG), β-1,4-N-acetylglucosaminidase (NAG), β-1,4-cellobiohydrolase (CBH), leucine aminopeptidase (LAP), and acid phosphatase (ACP));(b) PCA of total soil nutrient stoichiometric ratios (carbon-to-nitrogen ratio ($N_{C:N}$), carbon-to-phosphorus ratio ($N_{C:P}$), nitrogen-to-phosphorus ratio ($N_{N:P}$), carbon-to-nitrogen-to-phosphorus ratio ($N_{C:N:P}$)), microbial biomass C-N-P stoichiometric ratios (carbon-to-nitrogen ratio ($M_{C:N}$), carbon-to-phosphorus ratio ($M_{C:P}$), nitrogen-to-phosphorus ratio ($M_{N:P}$), carbon-to-nitrogen-to-phosphorus ratio ($M_{C:N:P}$)), and stoichiometric ratios of C-N-P-related enzyme activities (β-1,4-glucosidase (BG), β-1,4-N-acetylglucosaminidase (NAG), β-1,4-cellobiohydrolase (CBH), leucine aminopeptidase (LAP), and acid phosphatase (ACP)) (carbon-to-nitrogen ratio ($E_{C:N}$), carbon-to-phosphorus ratio ($E_{C:P}$), nitrogen-to-phosphorus ratio ($E_{N:P}$), carbon-to-nitrogen-to-phosphorus ratio ($E_{C:N:P}$)).
